# Is ^18^F-FDG PET/CT Beneficial for Newly Diagnosed Breast Cancer Patients With Low Proportion of ER Expression?

**DOI:** 10.3389/fonc.2021.755899

**Published:** 2021-11-04

**Authors:** Jiachen Liu, Runlu Sun, Yuping Yin, Jingyan Li, Xuming Liu, Sheng Liu, Zhanlei Zhang, Jieting Hu, Xiaoting Wan, Hong Zhang

**Affiliations:** ^1^ Department of Nuclear Medicine, Sun Yat-sen Memorial Hospital, Sun Yat-sen University, Guangzhou, China; ^2^ Department of Cardiology, Sun Yat-sen Memorial Hospital, Sun Yat-sen University, Guangzhou, China; ^3^ Department of Pathology, Sun Yat-sen Memorial Hospital, Sun Yat-sen University, Guangzhou, China

**Keywords:** ^18^F-FDG PET/CT, breast cancer, receptor status, the proportion of ER expression, the proportion of PR expression

## Abstract

**Objective:**

It is unclear whether the receptor status of breast malignancy or the proportion of receptors expression is useful in the interpretation of ^18^F-FDG PET/CT. This study’s purpose was to analyze whether ^18^F-FDG PET/CT was valuable for helping newly diagnosed breast cancer patients find suspected or unsuspected metastasis lesions based on the proportion of receptors expression.

**Materials and Methods:**

Eighty newly diagnosed breast cancer patients were divided into six groups, containing N0 (no extraaxillary lymph node metastasis), N1 (extraaxillary lymph node metastasis), M0 (no distant metastasis), and M1 (distant metastasis) groups, C0 (no unsuspected metastasis), and C1 (unsuspected metastasis and treatment plan changed) detected by PET/CT. The main data, including the proportion of receptors ER (estrogen receptor), PR (progesterone receptor), and Her-2 (human epidermal growth factor receptor 2) status, were extracted. Simple correlation and logistic regression were preformed to analyze the association between them.

**Results:**

Patients in N1 group had lower proportion of ER (%) and PR (%) than that in N0 group (ER: 2 [0–80] *vs.* 80 [15–95]; PR: 1 [0–10] *vs.* 20 [0–45], *p*<0.001). Moreover, the proportions of ER and PR were negatively correlated with N1 (ER: [r= −0.339, *p*= 0.002], PR: [r= −0.247, *p*= 0.011]) by simple correlation. Also, patients in C1 group had lower proportion of ER (%) and PR (%) than those in C0 group (ER: 10 [0–85] *vs.* 80 [15–90], *p*=0.026; PR: 1 [0–10] *vs.* 20 [0–70], *p*=0.041), while the distribution of ER and PR between M1 and M0 group had no significant difference. After the adjustment of traditional factors, the negative correlation between the proportion of ER (OR=0.986, 95% CI of OR [0.972–0.999], p=0.016) and C1 was found by logistic regression, cutoff value was 25% (ER) calculated by ROC (Receiver Operating Characteristic) curve (AUC [Area Under Curve]= 0.647, *p*=0.024).

**Conclusion:**

The proportion of ER in newly diagnosed breast cancer was negatively correlated with unsuspected metastasis detected by ^18^F-FDG PET/CT. ^18^F-FDG PET/CT might be recommended for newly diagnosed breast cancer patients with single lesions when the ER expression proportion is less than 25% to find unsuspected metastasis lesions and to modify treatment plan contrasted with conventional imaging and clinical examination.

## 1 Introduction

Recently, the International Agency for Research on Cancer (IARC) of the World Health Organization released the latest global cancer burden data for 2020, stating that breast cancer has replaced lung cancer and became the most prevalent cancer in the world (1). Early diagnosis and treatment of breast cancer has played a very important role in the fight against breast cancer (2).

^18^Fluorine-fluorodeoxyglucose (^18^F-FDG) positron emission tomography–computed tomography (PET/CT) is an imaging examination method for breast cancer and has become more important (3–5). It plays an important role in the systematic staging of breast cancer, detecting the effect of treatment, monitoring regeneration, and so on (2). National Comprehensive Cancer Network(NCCN)guidelines 2021 mentioned that ^18^F-FDG PET/CT may be helpful in identifying unsuspected regional nodal disease and/or distant metastases, and ^18^F-FDG PET/CT is most helpful in situations where standard staging studies are equivocal or suspicious, especially in the setting of locally advanced or metastatic disease (6). Meanwhile, PET/CT is a radiological and high-cost examination, and the application is also subject to certain restrictions (7). Although it is not suitable for all breast cancer patients, the use of ^18^F-FDG PET/CT may have important clinical effects for some appropriate patients (8).

Many previous studies on affecting the interpretation of ^18^F-FDG PET/CT in breast cancer mainly focused on the initial staging, subtypes, tumor grade, histologic types, and so on (4, 8, 9). Although these studies have achieved a lot of significant findings, there is considerable overlap between subtypes, and to date, no studies to my knowledge have shown the usefulness of receptor (ER [estrogen receptor], PR [progesterone receptor]) status of breast malignancy in the interpretation of ^18^F-FDG PET/CT, not to mention the proportion of the receptors expression in tumor cells. The receptor status of breast cancer has a very important influence on the progression of the disease, treatment methods, and prognosis (6, 10).Therefore, we performed this study to determine whether the proportion of receptors are related to the effectiveness of ^18^F-FDG PET/CT in helping newly diagnosed breast cancer patients to find extraaxillary lymph node metastasis and distant metastasis (suspected [suspected lesions were found by ultrasound, CT, and/or MR but not sure as metastasis] or unsuspected [unsuspected lesions were not found by ultrasound, CT, and/or MR before FDG PET/CT inspection]) and to help breast cancer patients get better diagnosis and treatment.

## 2 Article Types and Study Design

**Figure 1:**
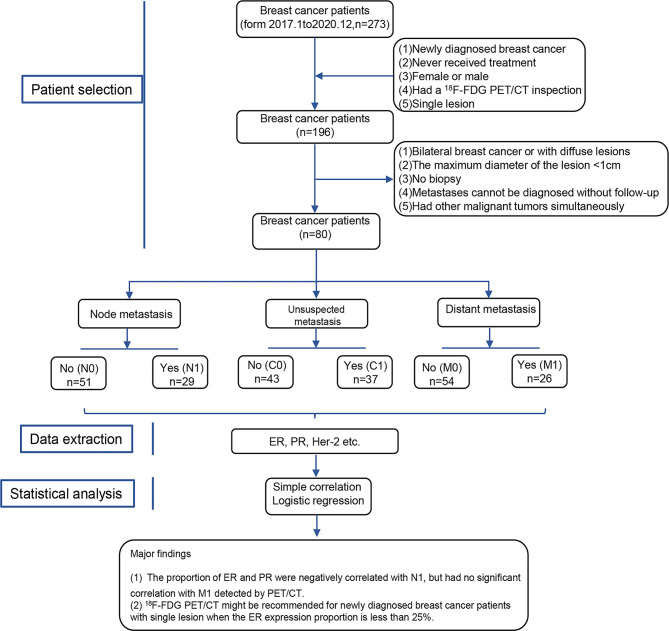
Workflow for this study design. N0, no extraaxillary lymph node metastasis; N1, extraaxillary lymph node metastasis; M0, no distant metastasis; M1, distant metastasis; C0, no unsuspected metastasis; C1, unsuspected metastasis and with initial treatment plan changed; ER, estrogen receptor; PR, progesterone receptor; Her-2, human epidermal growth factor receptor 2.

The study is a single-center retrospective cohort study. The overall research workflow is depicted in **Figure 1**, including patient selection, data extraction, statistical analysis, and conclusion. A brief description is as follows. We selected newly diagnosed breast cancer patients who underwent ^18^F-FDG PET/CT inspection based on the inclusion and exclusion criteria. These patients were divided into six groups, containing N0 (no extraaxillary lymph node metastasis), N1 (extraaxillary lymph node metastasis), M0 (no distant metastasis), M1 (distant metastasis), C0 (no unsuspected metastasis), and C1 (unsuspected metastasis and changed treatment plan) groups. The conventional factors (such as age or the maximum diameter of primary lesion), proportion of receptors (ER [estrogen receptor], PR [progesterone receptor]), and Her-2 (human epidermal growth factor receptor 2) status was extracted.

Next, the simple correlation and logistical regression analysis were used to determine the association between receptors, lymph node, and distant metastasis detected by ^18^F-FDG PET/CT. Finally, we drew a conclusion that the proportion of ER was negatively correlated with C1 detected by ^18^F-FDG PET/CT in newly diagnosed breast cancer patients. Expectantly, ^18^F-FDG PET/CT may be recommended for newly diagnosed breast cancer patients with single lesions when the proportion of ER was less than 25%.

## 3 Materials and Methods

### 3.1 Patient Selection

We selected a total of 196 newly diagnosed breast cancer patients who underwent ^18^F-FDG PET/CT inspection in our hospital from 2017 to 2020, excluded 116 cases, and at last included 80 cases for analysis. The inclusion criteria and exclusion criteria were as follows:

Inclusion criteria: (1) Newly diagnosed breast cancer. (2) Never received treatment. (3) Female or male. (4) Had a ^18^F-FDG PET/CT inspection. (5) Single lesion.

Exclusion criteria: (1) Bilateral breast cancer. (2) Diffuse lesions. (3) The maximum diameter of the lesion was less than 1 cm [NCCN guidelines and many other studies mentioned that the sensitivity and specificity of ^18^F-FDG PET/CT inspection are not high enough when faced with lesions with a maximum diameter of less than 1 cm (3, 4, 6, 8)]. (4) No biopsy. (5) Metastases cannot be diagnosed without follow-up. (6) Had other malignant tumors simultaneously.

### 3.2 Preparation Before Examination

The patient should rest 1 day in advance, avoid strenuous exercise to prevent muscle intake, fast for 4 h in advance, and forgo sugary fluids. On the examination day, the patient should drink 500 ml of water before the injection to dilute the ^18^F-FDG concentration in the urinary system, and keep warm to prevent brown fat from FDG avidity. The patient’s blood glucose level before examination needs to be kept <200 mg/dl to prevent the influence on ^18^F-FDG intake. Injection should be performed on the contralateral upper arm of the patient’s affected side to prevent the influence of human factors on the axillary lymph node metastasis of the affected side. Before the examination, the patient should urinate to excrete the higher concentration of FDG accumulated in the bladder. Imaging started 60 min after injection, and the range was generally from the skull to the mid-thighs of both sides.

### 3.3 Scan Parameters

Instrument model: Germany Siemens Biograph m CT·S. CT scan parameters: CT tube pressure 120 kv, tube current: 300MA, layer thickness 3–4 mm, matrix 512×512, radiopharmaceutical ^18^F-FDG, generated by GE800trace accelerator, imaging agent quality control radiochemical purity >95%. ^18^F-FDG was injected intravenously at 5.55 MBq/kg body weight. After the drug was injected, the patient should close their eyes and rest quietly for 40–60 min. PET/CT scan was performed after urination. Scan time: 15 min. The obtained images were transferred to the Medix workstation for reading and analysis after attenuation correction processing.

### 3.4 Immunohistochemistry

Tumor tissues resected intraoperatively were formalin-fixed and paraffin-embedded in time, cut into 3 μm sections, and baked at 68°C for 2 h. The tissue sections were stained immunohistochemically for ER, PR, and Her-2, respectively, using automated immunostainers (BOND-MAX; Leica Microsystems, Tokyo, Japan). The tissue sections were treated with biotinylated anti-rabbit secondary antibody, followed by further incubation with streptavidin-horseradish peroxidase complex. They were then stained with diaminobenzidine (DAB), and the sections were counterstained with hematoxylin.

### 3.5 Data Collection and Evaluation Criteria

#### 3.5.1 Data Collection

We collected the following patient information: traditional factors including age, gender, time (the time from the discovery of the abnormality, such as lump, pain, and so on, to the first visit to the doctor); histology (histological type including carcinoma *in situ*, infiltrating ductal carcinoma, infiltrating lobular carcinoma, and papillary carcinoma); grade (I, II, III); diameter (maximum diameter of the lesion); initial stage (staged by conventional imaging such as B ultrasonic, Computed Tomography and Magnetic Resonance Imaging and clinical examination; (staging standard is from the American Joint Committee on Cancer 8th edition); and proportion (positive expression in pathological tissue) of receptors including ER, PR, and Her-2 status. Then it was recorded whether the extraaxillary lymph node metastasis or distant metastasis (suspected or unsuspected) occurred for each case, and the changed treatment plan was recorded (breast-conserving surgery became full cut, surgery became neoadjuvant chemotherapy, and so on).

#### 3.5.2 Results of PET/CT Evaluation

A PET/CT report needs to be written by a senior nuclear medicine physician working for more than 5 years, and a nuclear medicine department associate deputy chief physician or chief physician should review it. If the result was uncertain, the department would discuss it to make a conclusion, and the specific receptor status would not be disclosed before the result is determined.

#### 3.5.3 Results of Pathology Evaluation

The results of immunohistochemical staining are evaluated by one experienced pathologist, and then reviewed by another experienced pathologist. In case of disagreement, a third party will re-evaluate or perform pathological re-examination.

### 3.6 Follow-Up

All the suspected or unsuspected extraaxillary nodal metastases and distant metastasis needed to be confirmed by pathological results. In addition, the lesion, such as small distant bone metastases, that cannot be confirmed by pathological results needed to be confirmed by more than two imaging methods during follow-up at least 3 months, and the stage finalized by the clinician and the corresponding treatment plan were recorded. The case follow-up was completed by a nuclear medicine physician and a non-nuclear medicine physician. When there was a disagreement, the opinion of the pathologist would be discussed and decided together.

### 3.7 Statistical Analysis

To compare continuous variables between groups, the statistical significance of differences was determined using Student’s *t* test or non-parametric test, as appropriate. Categorical variables were compared using the chi-square test. Simple correlation analysis and logistic regression analysis of the association between variables and N1, M1, and C1 after adjustment of potential confounders were performed. Finally, ROC (receiver operating characteristic) curve was used to test diagnostic power and determine the best cutoff value. The statistical software was SPSS 25. Statistical significance was defined as a two-tailed probability of less than 0.05.

## 4 Results

### 4.1 Accuracy Analysis of ^18^F-FDG PET/CT Inspection

Among all the 80 patients included in this research, there were three false positives (two related to lymph node metastasis were included in group N0 and C0 [sensitivity 93.55%], two related to distant metastasis was included in M0 and C0 [sensitivity 92.86%], there was one patient who overlapped) and three false negatives (two related to lymph node metastasis was included in group N1and C0 [specificity 96.23%], another one related to bone metastasis was included in group M1 and C0 [specificity 98.18%]). The total accuracy of ^18^F-FDG PET/CT inspection in this research was 93.02%.

### 4.2 Comparison of Baseline Characteristics and the Proportion of Receptors in Breast Cancer Patients With or Without Extraaxillary Lymph Node Metastasis Detected by ^18^F-FDG PET/CT Inspection

**Figure 2:**
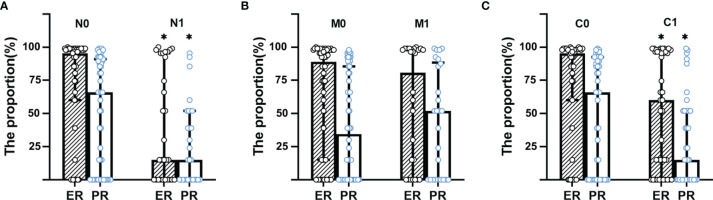
Box plot for the proportion of ER and PR distribution between groups. **(A)** Box plot for the proportion of ER and PR distribution between N group. **(B)** Box plot for the proportion of ER and PR distribution between M group. **(C)** Box plot for the proportion of ER and PR distribution between C group. N0, no extraaxillary lymph node metastasis; N1, extraaxillary lymph node metastasis; C0, no unsuspected metastasis; C1, unsuspected metastasis and with initial treatment plan changed; ER, estrogen receptor; PR, progesterone receptor. *p < 0.001.

The breast cancer patients with extraaxillary lymph node metastasis (N1) had lower proportion of the expression of ER (%) and PR (%) in tumor cells than N0 group (ER: 2 [0–80] *vs.* 80 [15–95]; PR: 1 [0–10] *vs.* 20 [0–45], *p*<0.001). Patients in N1group had higher diameter (mm, 40 [28–58] *vs.* 29 [21–42], *p*<0.001) than N0 group, as did initial stage (IIA [0, 0%], IIB [7, 24.14%], IIIA [13, 44.83%], IIIB [5, 17.24%], IIIC [2, 6.9%], IV [2, 6.9%] *vs.* IIA [11, 21.57%], IIB [24, 47.06%], IIIA [11, 21.57%], IIIB [3, 5.88%], IIIC [1, 1.96%], IV [1, 1.96%]). The time from the discovery of the abnormality, such as lump, pain, and so on, to the first visit to the doctor in N1 group was larger than that in N0 group (day, 150 [30–240] *vs.* 120 [15–365], *p*<0.001). Also, breast cancer patients with N1 had lower proportion of ER and PR by the box plots than that with N0 as shown in **Figure 2A**. Her-2 status, age, gender, histology, and grade were similar between N0 and N1 group in **Table 1** and **Supplementary Table 1** (*p*>0.05).

**Table 1:** Descriptive characteristics of included breast cancer patients grouped by extraaxillary lymph node metastasis, distant metastasis, and unsuspected metastasis.

	N0, n=51	N1, n=29	*p*	M0, n=54	M1, n=26	*p*	C0, n=43	C1, N=37	*p*
**Age, years**	47.86 ± 11.22	50.76 ± 8.81	0.265	**49 (41.5–56)**	**50 (41.75–56.75)**	**<0.001**	47.86 ± 10.64	50.41 ± 10.42	0.285
**Time, days**	**120 (15–365)**	**150 (30–240)**	**<0.001**	**90 (15–183)**	**183 (60–365)**	**<0.001**	**120 (15–365)**	**183 (30–365)**	**<0.001**
Unknown	**1 (1.96)**	**1 (3.45)**		**1 (1.85)**	**1 (3.85)**		**0 (0)**	**2 (5.41)**	
**Diameter, mm**	**29 (21–42)**	**40 (28–58)**	**<0.001**	**32 (22–42)**	**38 (25.25–61)**	**<0.001**	**27 (20.75–36.25)**	**42 (28.25–57.25)**	**<0.001**
**Initial stage**			**<0.001**			**<0.001**			**0.02**
IIA	**11 (21.57)**	**0 (0)**		**9 (16.67)**	**2 (7.69)**		**9 (20.93)**	**2 (5.41)**	
IIB	**24 (47.06)**	**7 (24.14)**		**22 (40.74)**	**9 (34.62)**		**19 (44.19)**	**12 (32.43)**	
IIIA	**11 (21.57)**	**13 (44.83)**		**17 (31.48)**	**7 (26.92)**		**9 (20.93)**	**15 (40.54)**	
IIIB	**3 (5.88)**	**5 (17.24)**		**3 (5.56)**	**5 (19.23)**		**2 (4.65)**	**6 (16.22)**	
IIIC	**1 (1.96)**	**2 (6.90)**		**3 (5.56)**	**0 (0)**		**1 (2.33)**	**2 (5.41)**	
IV	**1 (1.96)**	**2 (6.90)**		**0 (0)**	**3 (11.54)**		**3 (6.98)**	**0 (0)**	
**ER, %**	**80 (15–95)**	**2 (0–80)**	**<0.001**	60 (1–90)	50 (0–91.25)	0.909	**80 (15–90)**	**10 (0–85)**	**0.026**
**PR, %**	**20 (0–45)**	**1 (0–10)**	**<0.001**	4 (0-50)	10 (0–57.5)	0.580	**20 (0–70)**	**1 (0–10)**	**0.041**

Normally distributed continuous variables are presented as the mean ± standard deviation, while nonnormally distributed data are presented as the median (IQR, interquartile range). Categorical variables are presented as the number (percentage). N0, no extraaxillary lymph node metastasis; N1, extraaxillary lymph node metastasis; M0, no distant metastasis; M1, distant metastasis; C0, no unsuspected metastasis; C1, unsuspected metastasis and with initial treatment plan changed; Time, the time from the discovery of the abnormality (such as lump, pain, and so on) to the first visit to the doctor; Diameter, maximum diameter of the primary lesion; Initial stage, staged by conventional imaging (ultrasound, CT, and MR) and clinical examination (preliminary biopsy); ER, estrogen receptor; PR, progesterone receptor. P < 0.05 was considered significant and was bolded.

### 4.3 Comparison of Baseline Characteristics and the Proportion of Receptors in Breast Cancer Patients With or Without Distant Metastasis Detected by ^18^F-FDG PET/CT Inspection

The distribution of the proportion of ER and PR between M1 and M0 group had no significant difference as shown in **Figure 2B**, did nor did Her-2 status, gender, and grade in **Supplementary Table 1**. But age (year, 50 [41.75–56.75] *vs.* 49 [41.5–56], *p*<0.001), time (day, 183 [60–365] *vs.* 90 [15–183], *p*<0.001), diameter (mm, 38 [25.25–61] *vs.* 32 [22–42], *p*<0.001), and initial stage (IIA [2, 7.69%], IIB [9, 34.62%], IIIA [7, 26.92%], IIIB [5, 19.23%], IIIC [0, 0%], IV [3, 11.54%] *vs.* IIA [9, 16.67%], IIB [22, 40.74%], IIIA [17, 31.48%], IIIB [3, 5.56%], IIIC [3, 5.56%], IV [0, 0%]) in M1 group was higher than in M0. Furthermore, patients with the histological type of infiltrating ductal carcinoma had less distant metastasis (47 [83.93%] in M0 *vs.* 16 [66.67%] in M1, *p*<0.001), while patients with infiltrating lobular carcinoma had more distant metastasis (0 [0%] in M0 *vs.* 4 [16.67%] in M1, *p*<0.001) in **Supplementary Table 1**.

### 4.4 Comparison of Baseline Characteristics and the Proportion of Receptors in Breast Cancer Patients With or Without Unsuspected Metastasis (Extraaxillary Lymph Node Metastasis or Distant Metastasis, Which Led to Changing Treatment Plan) Detected by ^18^F-FDG PET/CT Inspection

The breast cancer patients with unsuspected metastasis found and treatment plan changed (C1) had lower proportion of the expression of ER (%) and PR (%) in tumor cells than the C0 group (ER: 10 [0–85] *vs.* 80 [15–90], *p*=0.026; PR: 1 [0–10] *vs.* 20 [0–70], *p*=0.041), as presented in **Figure 2C**. While patients in the C1 group had higher time (day, 183 [30–365] *vs.* 120 [15–365], *p*<0.001) and diameter (mm, 42 [28.25–57.25] *vs.* 27 [20.75–36.25], *p*<0.001) than in the C0 group. C1 group had more patients with IIIA, IIIB, IIIC initial stage than the C0 group (IIIA [15, 40.54%], IIIB [6, 16.22%], IIIC [2, 5.41%] *vs.* IIIA [9, 20.93%], IIIB [2, 4.65%], IIIC [1, 2.33%], *p*<0.001), while had fewer patients with IIA, IIB, IV initial stage than C0 group (IIA [2, 5.41%], IIB [12, 32.43%], IV [0, 0%] *vs.* IIA [9, 20.93%], IIB [19, 44.19%], IV [3, 6.98%]). Her-2 status, age, gender, histology, and grade were similar between C0 and C1 group in **Table 1** and **Supplementary Table 1** (*p*>0.05).

### 4.5 Association Between Receptors and N1, M1, C1 Detected by ^18^F-FDG PET/CT Inspection

Furthermore, the association between the receptors and extraaxillary lymph node metastasis or distant metastasis was analyzed by simple correlation. Data showed that the proportions of ER (r=−0.339, *p*=0.002) and PR (r=−0.247, *p*=0.011) were negatively correlated with the occurrence of extraaxillary lymph node metastasis (suspected and unsuspected), while diameter (r=0.3, *p*=0.008) and initial stage (r=0.455, *p*<0.001) were positively correlated with it, as presented in **Table 2A**. Similarly, the proportions of ER (r=−0.258, *p*=0.021) and PR (r=−0.217, *p*=0.054) were negatively correlated with the finding of unsuspected metastasis and a change in treatment plan. Diameter (r=0.399, *p*<0.001) and initial stage (r=0.262, *p*=0.019) were positively correlated with it, as presented in **Table 2C**. However, the proportion of ER (r=0.001, *p*=0.992) and PR (r=0.088, *p*=0.437) had no obvious correlation with distant metastasis, scilicet, no obvious correlation was found between the factors and the occurrence of distant metastasis, as shown in **Table 2B**.

**Table 2:** Association between ER, PR proportion, and extraaxillary lymph node metastasis or distant metastasis by simple correlation.

(A) Association between ER, PR proportion, and extraaxillary lymph node metastasis (suspected and unsuspected)			
	N	
	r	*P*	
**Time**	−0.39	0.734	
**Diameter**	**0.3**	**0.008**	
**Initial Stage**	0.455	**<0.001**	
**ER**	**−0.339**	**0.002**	
**PR**	**−0.247**	**0.011**	
**(B)** Association between ER, PR proportion, and distant metastasis (suspected and unsuspected)			
	**M**	
	**r**	***P***	
**Age**	0.31	0.787	
**Time**	0.219	0.054	
**Diameter**	0.214	0.065	
**Initial Stage**	0.209	0.062	
**ER**	0.001	0.992	
**PR**	0.088	0.437	
**(C)** Association between ER, PR proportion, and unsuspected metastasis with initial treatment plan changed.		
	**C**	
	**r**	***P***	
**Time**	0.097	0.400	
**Diameter**	**0.399**	**<0.001**	
**Initial Stage**	**0.262**	**0.019**	
**ER**	**−0.258**	**0.021**	
**PR**	−0.217	0.054	

N, extraaxillary lymph node metastasis; M, distant metastasis; C, unsuspected metastasis with initial treatment changed; r, correlation coefficient; Initial stage, staged by conventional imaging (ultrasound, CT, and MR) and clinical examination (preliminary biopsy); ER, estrogen receptor; PR, progesterone receptor. P < 0.05 was considered significant and was bolded.

Next, logistic regression was performed to analyze the correlation between the proportion of ER, PR, and the occurrence of extraaxillary lymph node metastasis, as in **Table 3A**. After adjustment of age, time, diameter, and initial stage, we found that the proportions of ER (OR=0.980, 95% CI of OR [0.964–0.996], *p*=0.013) and PR (OR=0.944, 95% CI of OR [0.909–0.980], *p*=0.003) were still negatively correlated with the occurrence of extraaxillary lymph node metastasis detected by ^18^F-FDG PET/CT, as did the time (OR=0.994, 95% CI of OR [0.990–0.998], *p*=0.002). And the positive correlation between age (OR=1.073, 95% CI of OR [1.003–1.149], *p*=0.042) and initial stage (OR=4.983, 95% CI of OR [2.152–11.537], *p*<0.001) between N1 also was found, as presented in **Table 3A**. The proportion of ER (OR=0.998, 95% CI of OR [0.973–1.024], *p*=0.890) and PR (OR=1.000, 95% CI of OR [0.972–1.029], *p*=0.988) had no obvious correlation with distant metastasis, nor did age and time. The maximum diameter of primary lesion (OR=1.037, 95% CI of OR [1.008–1.068], *p*=0.013) and initial stage (OR=1.617, 95% CI of OR [1.012–2.585], *p*=0.044) still had a positive correlation with M1, as shown in **Table 3B**. At last, after adjustment of the traditional factors, we found ER (OR=0.986, 95% CI of OR [0.972–0.999], *p*=0.039) had a negative correlation with the finding of unsuspected metastasis and treatment plan change. And the diameter (OR=1.058, 95% CI of OR [1.018–1.100], *p*=0.004) had a positive correlation with C1, as presented in **Table 3C**.

**Table 3:** Association between ER, PR, and N1, M1, C1 by logistic regression.

	B	SE	*p*	OR	95% CI of OR
**(A)** Association between ER, PR, and extraaxillary lymph node metastasis after adjustment of age, time, diameter, and initial stage.				
**ER**	**−0.020**	**0.008**	**0.013**	**0.980**	**0.964–0.996**
**PR**	**−0.058**	**0.019**	**0.003**	**0.944**	**0.909–0.980**
**Age**	**0.071**	**0.035**	**0.042**	**1.073**	**1.003–1.149**
**Time**	**−0.006**	**0.002**	**0.007**	**0.994**	**0.990–0.998**
**Diameter**	0.026	0.017	0.116	1.027	0.993–1.061
**Initial Stage**	**1.606**	**0.428**	**<0.001**	**4.983**	**2.152–11.537**
**(B)** Association between ER, PR, and distant metastasis after adjustment of age, time, diameter, and initial stage.				
**ER**	−0.002	0.013	0.890	0.998	0.973–1.024
**PR**	0.000	0.015	0.988	1.000	0.972–1.029
**Age**	0.037	0.028	0.431	0.1.022	0.968–1.079
**Time**	−0.001	0.001	0.380	0.999	0.998–1.001
**Diameter**	**0.037**	**0.015**	**0.013**	**1.037**	**1.008–1.068**
**Initial Stage**	**0.481**	**0.239**	**0.044**	**1.617**	**1.012–2.585**
**(C)** Association between ER, PR, and unsuspected metastasis with initial treatment changed after adjustment of age, time, diameter, and initial stage.				
**ER**	**−0.014**	**0.007**	**0.039**	**0.986**	**0.972–0.999**
**PR**	−0.018	0.009	0.061	0.982	0.965–1.001
**Age**	0.037	0.027	0.163	1.038	0.985–1.094
**Time**	−0.001	0.001	0.264	0.999	0.998–1.001
**Diameter**	**0.057**	**0.020**	**0.004**	**1.058**	**1.018–1.100**
**Initial Stage**	0.263	0.249	0.290	1.301	0.799–2.120

ER, estrogen receptor; PR, progesterone receptor; Time, the time from the discovery of the abnormality (such as lump, pain, and so on) to the first visit to the doctor; Diameter, maximum diameter of the primary lesion; Initial stage, staged by conventional imaging (ultrasound, CT, and MR) and clinical examination (preliminary biopsy). P < 0.05 was considered significant and was bolded.

**Figure 3:**
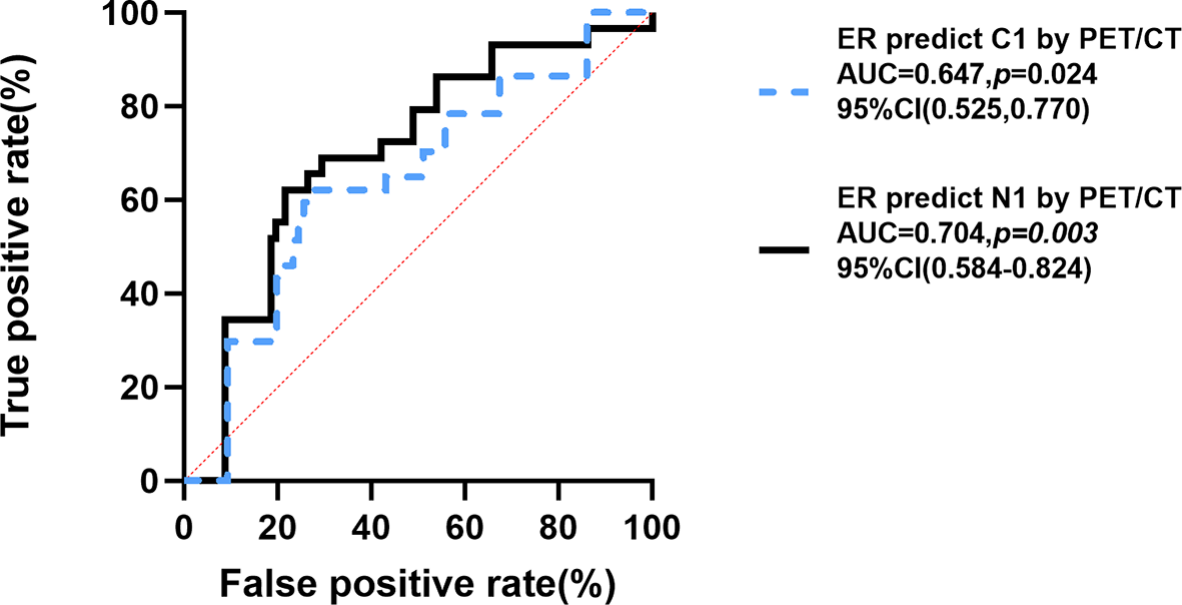
ROC curve for proportion of ER-positive expression predicting C1 and N1. Youden Index of N group and C group =25%. N1, extraaxillary lymph node metastasis; C0, no unsuspected metastasis; C1, unsuspected metastasis and with initial treatment plan changed; ER, estrogen receptor; ROC, Receiver Operating Characteristic; AUC, area under curve; CI, confidence interval. P < 0.05 was considered significant.

Finally, the receiver operating characteristic (ROC) curve was used to evaluate predictive values of the proportion of ER for N1 and C1 by ^18^F-FDG PET/CT inspection. **Figure 3** showed the area under the curve (AUC) for the relationships between the proportion of ER and N1 (AUC =0.704, 95% CI [0.584–0.824], *p*=0.003)and C1 (AUC=0.647, 95% CI [0.525–0.770], *p*=0.024). Youden index was calculated to obtain the best cutoff value, and all of N1 and C1 groups were 25% (the proportion of ER).

### 4.6 Detailed Sites of C1 Lesions Detected by ^18^F-FDG-PET Scans

There were 37 patients in C1 group. They had extraaxillary lymph node metastasis or distant metastasis lesions, which were ambiguous or neglected by conventional imaging (ultrasound, CT, and/or MRI) and clinical examination (lymph node biopsy) but identified by ^18^F-FDG-PET. The detailed sites of lesions that led to changing the treatment plan are shown in **Table 4**, *p*= 0.037<0.005 (chi-square test). There were 15 (40.54%) patients with clavicle area and/or inner mammary area lymph node metastases, 12 (32.43%) of which with the proportion of ER ≤ 25% (ER−), 3 (8.11%) with the proportion of ER>25% (ER+). Three (8.11%) patients had liver metastasis, two (5.41%) patients with ER−, and one (2.7%) patient with ER+. One (2.7%) patient had lung metastasis and with ER−. Seven (18.92%) patients had bone metastasis, one (2.7%) patient with ER−, and six (16.22%) patients with ER+. Eleven (29.72%) patients had multiple metastases, in which one (2.7%) patient had lung and bone metastases with ER−, two (5.41%) patients had lung, clavicle area, and/or inner mammary area lymph node metastases with ER−, eight (21.62%) patients had bone, clavicle area, and/or inner mammary area lymph node metastases (five [13.51%] patients with ER− and three [8.11%] patients with ER+).

**Table 4:** Detailed sites of lesions leading to changing the treatment plan in C1 group.

	N	Liver	Lung	Bone	Multiple metastases
ER−	12 (32.43)	2 (5.41)	1 (2.7)	1 (2.7)	8 (21.62)
ER+	3 (8.11)	1 (2.7)	0 (0)	6 (16.22)	3 (8.11)

Data are presented as n (%). ER−, the proportion of ER ≤ 25%; ER+, the proportion of ER>25%; N, patients with clavicle area and/or inner mammary area lymph node metastases. p= 0.037 (chi-square test).

### 4.7 Examples

**Figure 4:**
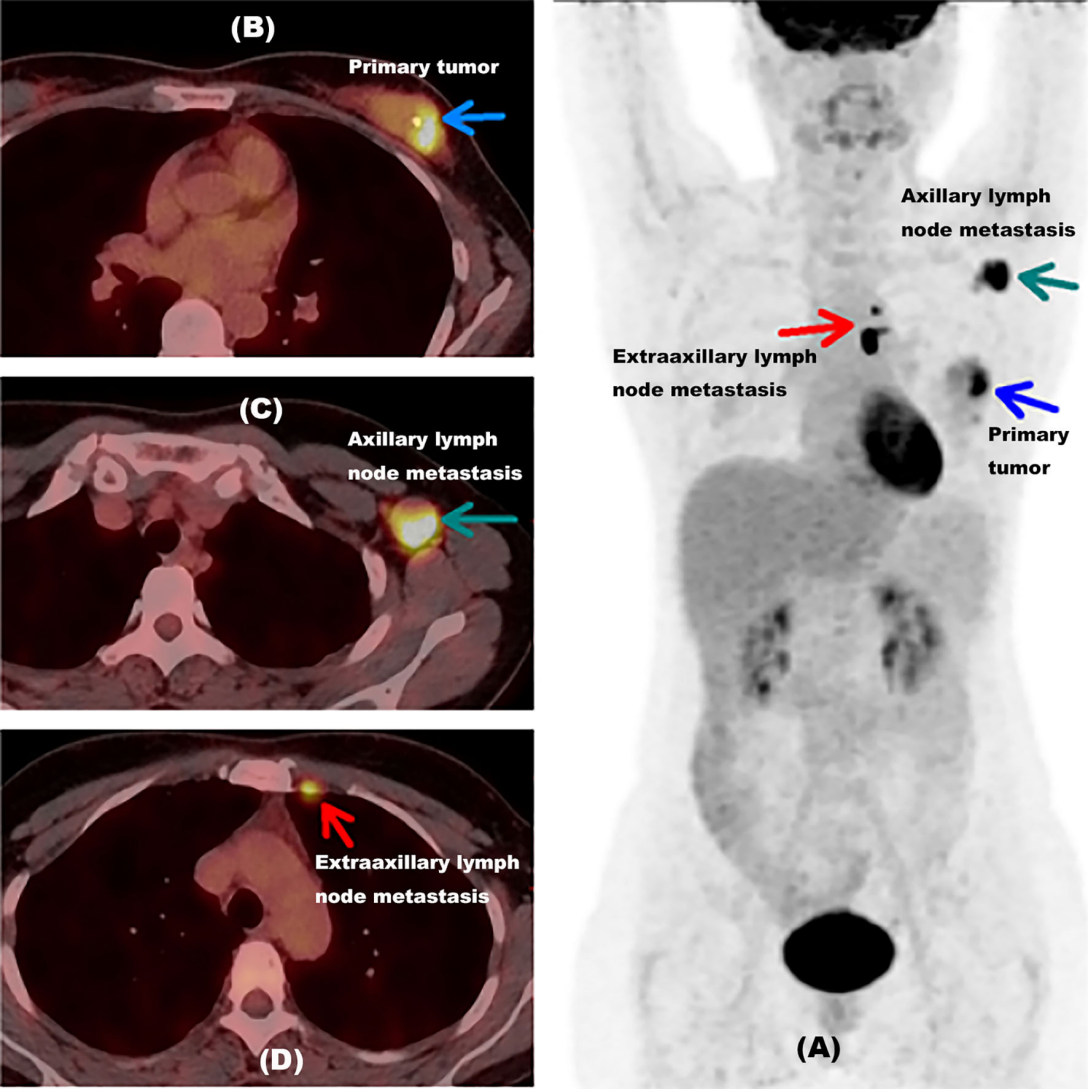
Example for a newly diagnosed breast cancer patient with unsuspected extraaxillary lymph node metastasis detected by 18F-FDG PET/CT inspection. **(A)** A MIP picture of a 48-year-old newly diagnosed breast cancer woman detected by an 18F-FDG PET/CT inspection with the proportion of ER at 1%. The blue arrow showed the primary tumor of this patient. The green arrow indicated axillary lymph node metastasis in the left armpit. The red arrow presented extraaxillary lymph node metastasis in the left internal mammary near breastbone. **(B)** PET/CT fusion image of this woman with primary tumor. The blue arrow showed the primary tumor of this patient. **(C)** PET/CT fusion image of this woman with axillary lymph node metastasis. The green arrow indicated axillary lymph node metastasis in the left armpit. **(D)** PET/CT fusion image of this woman with extraaxillary lymph node metastasis. The red arrow presented extraaxillary lymph node metastasis in the left internal mammary near the breastbone. ER, estrogen receptor; MIP, maximum intensity projection, with the proportion of ER lower than 25%.

**Figure 4** shows an example of a 48-year-old newly diagnosed breast cancer woman detected by ^18^F-FDG PET/CT inspection. Unsuspected extraaxillary lymph node metastasis in the left internal mammary near the breastbone was found by ^18^F-FDG PET/CT, which was neglected by MR. The ER proportion of this patient was 1% (less than 25%). This patient originally planned to undergo surgical treatment, but after the lesion was discovered by ^18^F-FDG PET/CT, the treatment plan was updated; this patient would take neoadjuvant chemotherapy first and then surgery. This example indicated that when the proportion of ER was less than 25%, ^18^F-FDG PET/CT might help newly diagnosed breast cancer patients find unsuspected extraaxillary lymph node metastasis and change the initial treatment plan.

**Figure 5:**
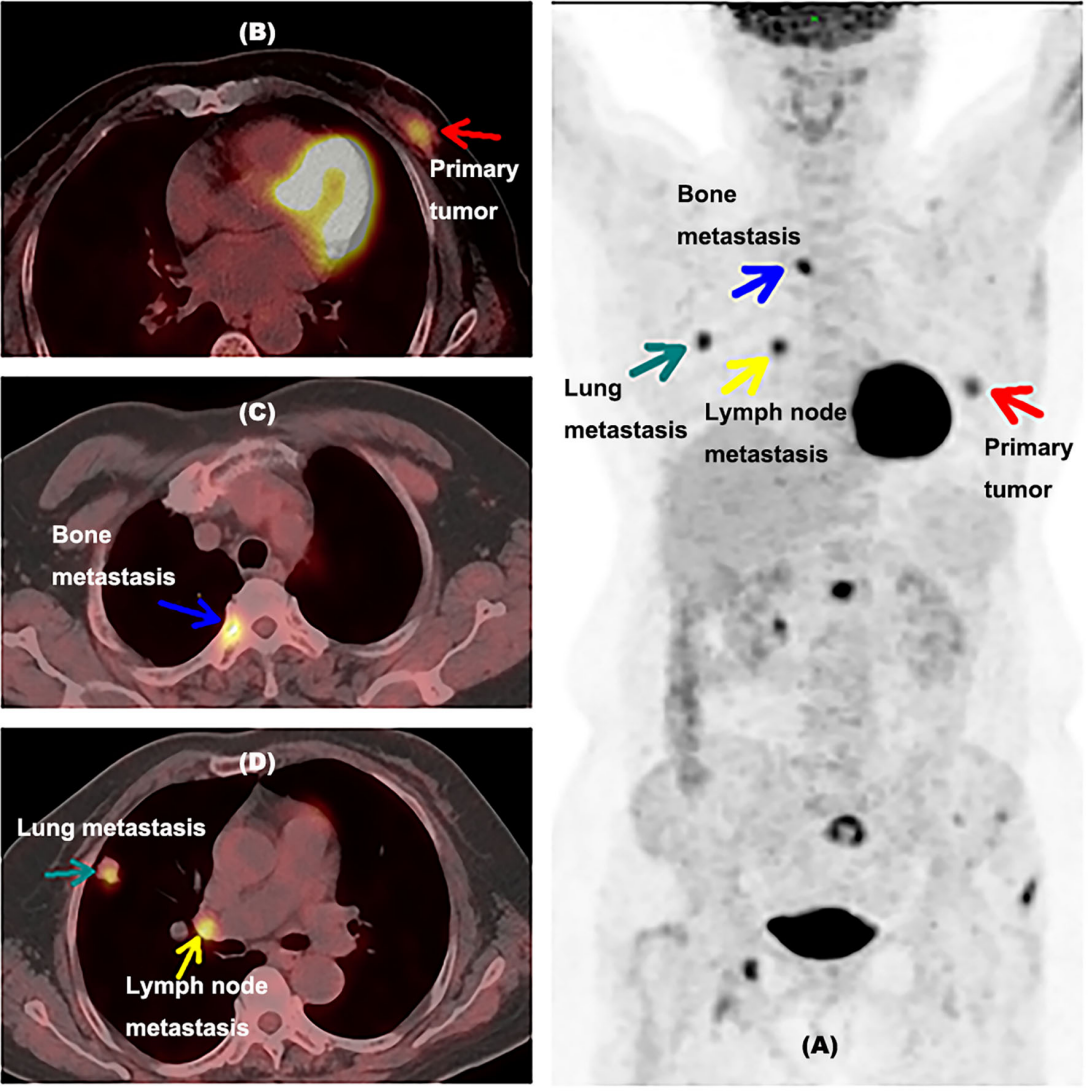
Example for a newly diagnosed breast cancer patient with unsuspected lung, hilar lymph node, and bone metastases detected by 18F-FDG PET/CT inspection. **(A)** A MIP picture of a 63-year-old newly diagnosed breast cancer woman detected by an ^18^F-FDG PET/CT inspection with the proportion of ER at 10%. The red arrow showed the primary tumor of this patient. The green arrow indicated lung metastasis in the right lung. The yellow arrow presented hilar lymph node metastasis. The blue arrow showed bone metastasis. **(B)** PET/CT fusion image of this woman with primary tumor. **(C)** PET/CT fusion image of this woman with lung metastasis and hilar lymph node metastasis. The green arrow indicated lung metastasis, and the yellow arrow presented hilar lymph node metastasis. **(D)** PET/CT fusion image of this woman with bone metastasis. ER, estrogen receptor; MIP, maximum intensity projection, with the proportion of ER lower than 25%.

**Figure 5** shows another example of a 63-year-old newly diagnosed breast cancer patient detected by ^18^F-FDG PET/CT inspection. There was no metastasis found except the primary lesion in initial diagnosis, but ^18^F-FDG PET/CT inspection was performed at the request of the patient, then unsuspected distant metastases in lung, hilar lymph node, and bone were detected, confirmed by needle biopsy, which were neglected by ultrasound and MRI. The treatment plan was updated to chemotherapy and targeted drug therapy. The ER proportion of this patient was 10% (less than 25%). This example indicated that although no axillary lymph node metastasis was found in the initial diagnosis, it is still possible to find unsuspected metastases by FDG PET/CT inspection when the ER was less than 25%.

## 5 Discussion

Intriguingly, it will be a novel strategy to perform ^18^F-FDG PET/CT for newly diagnosed breast cancer patients to find lymph node metastasis and distant metastasis based on their proportion of receptors expression in tumor cells. In this study, we found that (1) the proportion of ER and PR was negatively correlated with extraaxillary lymph node metastases (suspected or unsuspected). (2) The proportion of ER and PR had no significant correlation between distant metastasis (suspected or unsuspected). (3) The proportion of ER was related to the finding of unsuspected metastasis lesions contrasted with conventional imaging (ultrasound, CT, and MR) and clinical examination (preliminary biopsy). (4)^18^F-FDG PET/CT may be recommended for newly diagnosed breast cancer patients with single lesion when the ER marking index was less than 25%.

Many studies mentioned that ^18^F-FDG PET-CT had the limitation of diagnostic sensitivity and specificity for the primary breast tumor (11, 12). This is because the radioactive distribution of inflammation and granuloma would affect the differential diagnosis (13–15), and the detection efficiency of ^18^F-FDG PET-CT for axillary lymph node metastasis is lower than that of sentinel lymph node biopsy (16). Yet for locoregional extraaxillary nodes including internal mammary, infraclavicular, and supraclavicular nodes, ^18^F-FDG PET-CT has obvious advantages in the detection of these lymph node metastases (17–19), as our finding shows. Similarly, for distant metastases such as brain, lung, liver, and bone, ^18^F-FDG PET-CT examination has obvious advantages over other examination methods and contributes to improving staging and treatment (20). And the NCCN guideline of breast cancer also expressed similar views (17, 20, 21).

Some previous studies had found that breast cancer patients with negative ER expression were more likely to have metastases (10). Deborah Smith et al. mentioned that high expression of ER can prevent the occurrence of lymph node metastasis (22), which is similar to the results of our study. ER is a transcription factor that regulates gene expression events that ultimately lead to cell division, and this important property contributes to its key role in breast development (23). In this study, we found that the proportion of ER of breast cancer patients had a negative correlation with the occurrence of extraaxillary lymph node metastasis. ER coordinates the initiation of cell division during breast development and post-pubertal physiological functions (such as pregnancy), and has a synergistic effect with other hormones and their nuclear receptor transcription factors (including progesterone and prolactin) (24). The ability of ER to associate with DNA and initiate gene transcription has been subverted in diseases, where ER becomes a driving transcription factor that is no longer regulated by a control mechanism, which leads to estrogen-induced tumors. Essentially, ER continues to function as a gene that regulates transcription factors, but ER-mediated cell division occurs in an uncontrolled manner, leading to tumorigenesis and cancer progression (25, 26).

Progesterone receptor (PR) is the main regulator in female reproductive tissues, which can control the development process, proliferation, and differentiation during the reproductive cycle and pregnancy (27). PR also plays a role in the progression of endocrine-dependent breast cancer (24). As a member of the ligand-dependent transcription factor nuclear receptor family, the main role of PR is to regulate the network of target gene expression and respond to its associated steroid hormone and progesterone reaction (28–30). Some studies suggested that ER and PR have a synergistic effect (10, 22). In this study, we found the proportions of ER and PR were negatively correlated with N1 but had no significant correlation with M1 (16 patients with bone metastasis in 26 patients with distant metastasis) by simple correlation. It was consistent with some studies declaring that breast cancer patients with ER (+) in tumor cells have higher rates of bone metastasis compared to patients with ER (−), while breast cancer patients with ER (−) in tumor cells have higher rates of other organs (such as brain, lung, liver) metastasis compared to patients with ER (+) (31, 32), and this may affect the effectiveness of the proportion of ER in predicting unsuspected metastasis lesions in the whole body (N group AUC=0.704 *vs.* C group AUC=0.647). The potential molecular mechanism might be the differential expression of ER-target genes, which leads to the involvement of transforming growth factor β and fibroblast growth factor (FGF) signaling, TFF proteins, IL11, and CTGF in bone metastases. And breast cancer patients with ER (−) in tumor cells having higher rates of other organs (such as brain, lung, liver) metastasis might relate to the downregulated mammaglobin and lipophilin B, which are located in the 11q13 (33), so as to let ^18^F-FDG PET/CT play a greater role in finding metastases and improving staging in the low ER expression group.

In addition, malignant cells are known to have accelerated metabolism, high glucose requirements, and increased glucose uptake (34). Increased glucose transporters in malignant cells has been associated with increased and deregulated expression of glucose transporter proteins (GLUT1 and/or GLUT3). The study by P Laudański et al. showed that most ER-alpha-negative were GLUT1 positive, and significant correlation exists between the two receptors (35), which might lead to the metastases lesions being easiet to detect by ^18^F-FDG PET/CT.

Also, we found that the maximum diameter in newly diagnosed breast cancer patients with single primary lesions was positively correlated with the finding of unsuspected metastasis (36, 37), which was similar to some previous studies.

## 6 Strength and Limitation

We found when the proportion of ER is less than 25%, ^18^F-FDG PET/CT may be better at detecting unsuspected extraaxillary lymph node metastasis in breast cancer patients and changing the initial treatment plan, so as to help ^18^F-FDG PET/CT get better application and help breast cancer patients get better diagnosis and treatment. However, the expression of Her-2 in this study was not statistically relevant to the occurrence of extraaxillary lymph node metastasis or distant metastasis through ^18^F-FDG PET/CT inspection. The reason may be that the sample size was not large enough, or there was a synergistic effect between the factors. These still need to be further expanded by subsequent research work and more precise study.

## Data Availability Statement

The raw data supporting the conclusions of this article will be made available by the authors, without undue reservation.

## Ethics Statement

The studies involving human participants were reviewed and approved by Sun Yat-sen Memorial Hospital Ethics Committee. Written informed consent for participation was not required for this study in accordance with the national legislation and the institutional requirements.

## Author Contributions

HZ, RS, and JCL conceived of, designed, and supervised the study. JCL and RS wrote the manuscript. YY, JYL, and XL collected and analyzed the data. SL, ZZ, JH, and XW provided technical assistance with the study. All authors contributed to the article and approved the submitted version.

## Funding

This study was supported by grants from the Natural Science Foundation of China (Grant number: 81700397).

## Conflict of Interest

The authors declare that the research was conducted in the absence of any commercial or financial relationships that could be construed as a potential conflict of interest.

## Publisher’s Note

All claims expressed in this article are solely those of the authors and do not necessarily represent those of their affiliated organizations, or those of the publisher, the editors and the reviewers. Any product that may be evaluated in this article, or claim that may be made by its manufacturer, is not guaranteed or endorsed by the publisher.

## Supplementary Material

Supplementary Table 1 Descriptive characteristics of included breast cancer patients grouped by extraaxillary lymph node metastasis, distant metastasis, and unsuspected metastasis.
