# Supplementary material for: Is 18F-FDG PET/CT Beneficial for Newly Diagnosed Breast Cancer Patients With Low Proportion of ER Expression?
Source: Front Oncol. 2021 Nov 4;11:755899. doi: 10.3389/fonc.2021.755899 (PMC8599817; doi:10.3389/fonc.2021.755899)
Supplement: Supplementary Table 1 — Descriptive characteristics of included breast cancer patients grouped by extraaxillary lymph node metastasis, distant metastasis, and unsuspected metastasis. [file Table_1.docx]

Supplementary Material

**Supplementary Table 1.** Descriptive characteristics of included breast cancer patients grouped by extraaxillary lymph node metastasis，distant metastasis and unsuspected metastasis.

|  | **N0, n=51** | **N1, n=29** | ***p*** | **M0, n=54** | **M1, n=26** | ***p*** | **C0, n=43** | **C1, N=37** | ***p*** |
| --- | --- | --- | --- | --- | --- | --- | --- | --- | --- |
| **Histology** |  |  | *0.053* |  |  | *＜0.001* |  |  | *0.250* |
| **Carcinoma** |  |  |  |  |  |  |  |  |  |
| **in situ** | 1（1.96） | 1（3.45） |  | 2（3.70） | 0（0） |  | 1（2.32） | 1（2.7） |  |
| **IDC** | 41（80.39） | 22（75.86） |  | 47（87.04） | 16（61.54） |  | 36（83.72） | 27（72.97） |  |
| **ILC** | 5（9.80） | 0（0） |  | 0（0） | 4（15.38） |  | 1（2.32） | 4（10.81） |  |
| **Papillary** | 1（1.96） | 1（3.45） |  | 1（1.85） | 1（3.85） |  | 1（2.33） | 1（2.7） |  |
| **Unknown** | 3（5.88） | 5（17.24） |  | 4（7.41） | 5（19.23） |  | 4（9.3） | 4（10.81） |  |
| **Grade** |  |  | *0.872* |  |  | *0.548* |  |  | *0.584* |
| **I** | 0（0） | 0（0） |  | 0（0） | 0（0） |  | 0（0） | 0（0） |  |
| **II** | 17（33.33） | 8（27.59） |  | 18（33.33） | 7（29.17） |  | 16（37.21） | 9（20.93） |  |
| **III** | 20（39.22） | 10（34.48） |  | 23（42.59） | 7（29.17） |  | 17（39.53） | 13（30.23） |  |
| **Unknown** | 14（27.45） | 11（37.93） |  | 13（24.07） | 12（41.67） |  | 10（23.26） | 15（34.88） |  |
| **Female** | 50（98.00） | 29（100） | *1.000* | 53（98.15） | 26（100.00） | *1.000* | 42（97.67） | 37（100） | *1.000* |
| **Her-2** |  |  | *0.444* |  |  | *0.239* |  |  | *0.250* |
| **-** | 2（3.92） | 2（6.90） |  | 2（3.70） | 2（7.69） |  | 1（2.33） | 3（8.11） |  |
| **+** | 48（94.12） | 26（89.66） |  | 51（94.44） | 23（88.46） |  | 41（95.35） | 33（89.19） |  |
| **Unknown** | 1（1.96） | 1（3.45） |  | 1（1.85） | 1（3.85） |  | 1（2.33） | 1（2.7） |  |

Normally distributed continuous variables are presented as the mean ± standard deviation, while nonnormally distributed data are presented as the median (IQR, interquartile range). Categorical variables are presented as the number(percentage). N0 = no extraaxillary lymph node metastasis, N1 = extraaxillary lymph node metastasis, M0 = no distant metastasis, M1 = distant metastasis, C0=no unsuspected metastasis, C1=unsuspected metastasis and with initial treatment plan changed, IDC = infiltrating ductal carcinoma, ILC = infiltrating lobular carcinoma, Her-2 = human epidermal growth factor receptor 2.
